# Differential gene expression and SNP association between fast- and slow-growing turbot (*Scophthalmus maximus*)

**DOI:** 10.1038/s41598-017-12459-4

**Published:** 2017-09-21

**Authors:** Diego Robledo, Juan A. Rubiolo, Santiago Cabaleiro, Paulino Martínez, Carmen Bouza

**Affiliations:** 1 Departamento de Zooloxía, Xenética e Antropoloxía Física, Facultade de Veterinaria, Universidade de Santiago de Compostela, 27002 Lugo, Spain; 2 The Roslin Institute and Royal (Dick) School of Veterinary Studies, University of Edinburgh, Midlothian, EH25 9RG United Kingdom; 3 Cluster de Acuicultura de Galicia (Punta do Couso), Aguiño-Ribeira, 15695 Spain

## Abstract

Growth is among the most important traits for animal breeding. Understanding the mechanisms underlying growth differences between individuals can contribute to improving growth rates through more efficient breeding schemes. Here, we report a transcriptomic study in muscle and brain of fast- and slow-growing turbot (*Scophthalmus maximus*), a relevant flatfish in European and Asian aquaculture. Gene expression and allelic association between the two groups were explored. Up-regulation of the anaerobic glycolytic pathway in the muscle of fast-growing fish was observed, indicating a higher metabolic rate of white muscle. Brain expression differences were smaller and not associated with major growth-related genes, but with regulation of feeding-related sensory pathways. Further, SNP variants showing frequency differences between fast- and slow-growing fish pointed to genomic regions likely involved in growth regulation, and three of them were individually validated through SNP typing. Although different mechanisms appear to explain growth differences among families, general mechanisms seem also to be involved, and thus, results provide a set of useful candidate genes and markers to be evaluated for more efficient growth breeding programs and to perform comparative genomic studies of growth in fish and vertebrates.

## Introduction

Growth, the main target of aquaculture breeding programs^1^, is a complex polygenic trait with high environmental influence. Fast and efficient growth shortens the time required to reach market size, increases food conversion efficiency, and, in sum, leads to higher profitability and food safety. Aquaculture is currently the fastest growing food industry^2^; however, despite its importance, genetic breeding programs are applied in no more than 10% of cultured species^1,3^.

Growth is a trait of moderate to high heritability^3^ that displays a complex development and physiology. It involves both the increase of muscle cell number (hyperplasia) and cell size (hypertrophy), and the balance between both processes depends on the developmental stage, but it is also influenced by the environment. Growth is regulated by the hypothalamic-pituitary axis hormones which also control feeding behaviour^4^, yet local interactions between different muscle cell types and with other nearby tissues also condition muscle growth. Additionally, growth rate is regulated by other physiological and environmental factors, such as food intake, temperature, diet composition, age or sex^5^. Furthermore, identifying key growth-related genes is more complex in teleost than in other vertebrates, since this group of fish has undergone an additional round of genomic duplication^6^ involving neo- or sub-functionalization processes. Yet, although a complex task, understanding the genetic basis of growth variation might aid to achieve more efficient breeding programs. Despite its good response to traditional selection, the sole application of phenotypic and relatedness information to improve growth rate might determine the loss of relevant genetic variation affecting the medium- and long-term performance of breeding programs^7,8^. In this sense, knowing the molecular mechanisms, genes and pathways involved in growth can have multiple benefits. Beyond increasing our basic knowledge with important implications in different research areas related to growth, identification of allelic variants of moderate to large effect could be useful in marker-assisted selection (MAS), provided a cost-benefit balance. Finally, genome-editing techniques may allow recovering or even introducing variants not present in the population of interest, increasing genetic gain^9^. As such, different studies have been focused on understanding growth differences in different fish species in the last decade^10–15^.

Turbot is the flatfish with highest aquaculture production, mainly held by Spain and China. Females reach sexual maturity at ~3 years (~50 cM length) and can spawn between 5 and 10 million eggs per season. Reproduction can be achieved along the whole year under a variable controlled photoperiod^16^. Thus, production can benefit from the high fecundity of a single selected couple over a relatively long period. Several studies have been carried out in this species to identify quantitative trait loci (QTL) associated with growth^17–19^, but the information provided, though useful, is still of limited application. Knowing the genes or even the allelic variants behind growth differences is important, not only for a more efficient selection, but also to guide future studies in turbot and other fish species. However, functional studies on differential growth have not been performed in turbot yet.

To gain knowledge on the key genes involved in growth, we carried out a transcriptomic study in fast- and slow-growing turbot to identify differential expression profiles and genetic markers between extreme growth phenotypes. Samples of muscle and brain tissues were taken from family-matched fast- and slow-growth fish selected from eight unrelated full-sib families, and their transcriptomes compared by RNA-Seq. These organs play a key role in the regulation of somatic growth, and muscle also represents the main edible part of fish^5,20^. In order to evaluate a high number of individuals and to avoid family bias, a pooling strategy was followed. Differential gene expression and marker association between high- and low-growth turbot were obtained. The detected candidate genes and markers were mapped using the recently assembled genome of the species and their positions were compared with previously reported QTLs^21^. The most interesting genes and markers were validated using individual Real-Time PCR (qPCR) and SNP genotyping to disentangle general and family-specific factors involved in turbot growth.

## Results

### RNA sequencing output

A total of ~183 million paired-end (PE) reads were generated, for an average of ~15 million reads for each of the twelve samples analyzed. After filtering, ~171 million reads were retained (93.44%; >13 million reads per sample). On average, 88.4% of the PE filtered reads aligned to the turbot genome at unique (83.9%) or multiple (4.5%) genomic positions, whereas the remaining 11.6% did not match. The resulting muscle and brain transcriptomes revealed a total of 30,674 and 49,034 transcripts, respectively, belonging to 17,173 and 23,104 genes (Table 1), which included some non-annotated genes in the turbot genome^21^. Annotation against NCBI’s non redundant protein database was successful for 87.2% and 86.7% of the muscle and brain transcriptomes, respectively.

**Table 1:** Muscle and brain transcriptome statistics.

	Muscle	Brain
Number of transcripts	30,674	49,034
Number of genes	17,173	23,104
N50	3,734 bp	5,062 bp
N90	1,517 bp	2,122 bp
Total transcripts >500 bp	29,901 (97.5%)	47,517 (96.9%)
Average transcript length	2,859 bp	3,788 bp

### Differential gene expression

**Figure 1:**
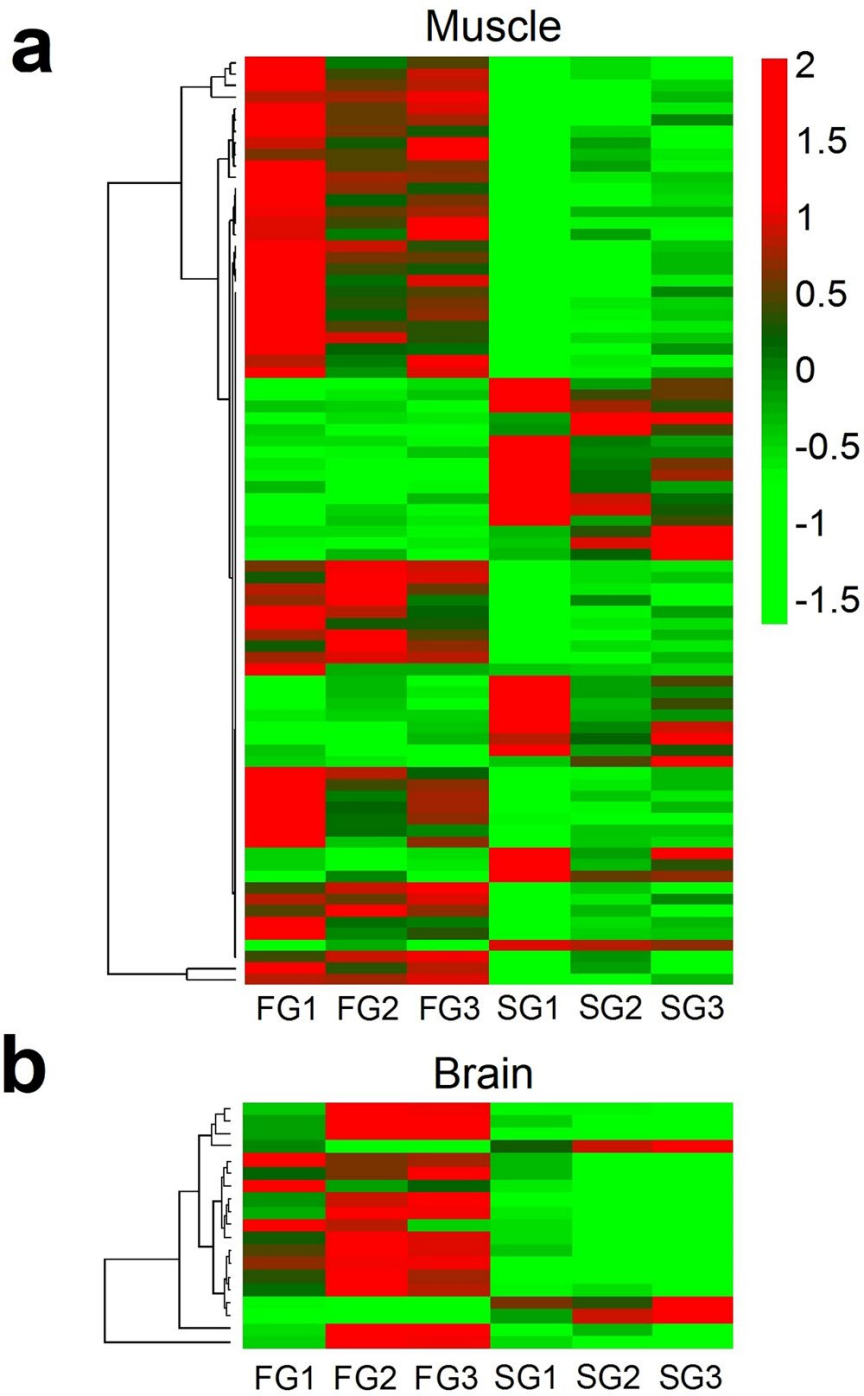
Muscle and brain heatmaps. Heatmaps of genes showing FDR corrected p-values < 0.3 for fast- (FG) and slow- (SG) growing turbot in: (**a**) muscle and (**b**) brain. Displayed are DESeq. 2 v.3.2 (Love *et al*.^59^) normalized counts for each sample and gene scaled by gene. Genes were hierarchically clustered according to their gene expression using Pearson correlation as a distance measure.

A total of 17 and 4 differentially expressed (DE) genes were detected in muscle and brain, respectively, when comparing fast- (FG) and slow- (SG) growing pooled samples using the log2 FG/SG fold change (FC) (Table 2). FC values were moderate, ranging from 1.18 to −0.86 FC values, positive FC corresponding to higher expression in FG compared to SG (up-regulated onwards), and negative FC to lower expression in FG compared to SG (down-regulated onwards). Since the number of DE genes was low, an extended list of those genes with FDR < 0.3 was also explored (Supplementary Dataset 1). Overall expression patterns were consistent across the three biological pools tested (Fig. 1), especially considering that FG and SG pools belong to eight different families and that the mechanisms driving growth differences are not necessarily the same for all the families. No significant sex-ratio differences between pools and families were found, supporting that sex was not a confounding factor in this study. KEGG enrichment analysis revealed the prevalence of certain pathways among the extended list of DE genes. The pathways consistently enriched were “Glycolysis/Gluconeogenesis” and “Starch and sucrose metabolism” (log_2_ fold enrichment of 5.3 and 4.8, respectively) (Supplementary Figure 1), involving a set of 16 up-regulated genes coding for enzymes of the glycolytic pathway.

**Table 2:** Differentially expressed genes between fast- and slow-growing turbot.

Gene	FDR p-val	FC	LG	Position
**Muscle**				
Triosephosphate isomerase	0.001	0.79	LG02	13,361,150
Glucose-6-phosphate isomerase	0.004	0.77	LG04	21,441,582
Beta-enolase	0.008	0.77	LG16	147,95,147
C-binding protein	0.015	1.13	LG10	17,180,345
Glyceraldehyde-3-phosphate dehydrogenase	0.016	0.72	LG02	13,180,325
Phosphoglycerate kinase 1	0.016	0.72	LG08	14,493,171
Fish-egg lectin	0.016	0.99	UN	UN
Myosin heavy fast skeletal muscle	0.016	0.75	LG06	19,857,385
Extensin-2	0.017	1.18	LG09	14,700,577
Mesothelin	0.023	1.10	LG03	14,490,203
Fructose-bisphosphate aldolase a	0.024	0.67	LG21	9,095,011
Flocculation protein flo11	0.027	0.80	LG01	3,075,171
Type ii cytoskeletal 8	0.037	0.95	LG10	18,632,403
Gelsolin	0.037	0.96	LG05	15,564,429
Protein-glutamine gamma-glutamyltransferase e	0.037	0.98	LG05	10,093,262
Thymosin beta-a	0.044	0.87	LG07	7,429,705
Chitin synthase 2	0.044	1.03	LG22	917,070
**Brain**				
Non annotated transcript	0.000	−0.86	LG03	12,013,135
Histone-lysine n-methyltransferase	0.005	0.60	LG22	1,690,914
Hyaluronan and proteoglycan link protein 1	0.039	0.60	LG12	959,526
Synaptoporin	0.039	0.66	LG11	5,169,652

FDR p-val: false discovery rate corrected p-value; FC: log2 fold change; LG: linkage group; UN: gene located in a scaffold not assigned to a LG of the turbot genetic map.

Among other interesting up-regulated genes in muscle, we detected six myosin heavy chain genes (FDR p-values ranging from 0.016 to 0.295 and FC from 1.00 to 0.48), strongly related to muscle growth; and the elongation factor 1-alpha (p-val = 0.099, FC = 0,70), which suggests enhanced protein synthesis for muscle growth. Interestingly, growth hormone receptor 2 (GHR2) was down-regulated (p-val = 0.063, FC = −1.01), showing an opposite pattern to that expected according to the function of the growth hormone receptor in mammals. Furthermore, the cytosolic creatine kinase (CKs) was up-regulated (p-val = 0.081, FC = 0.59), while its mitochondrial counterpart (CKm) was down-regulated (p-val = 0.170, FC = −0.67), in agreement with the higher production of cytosolic ATP due to the activation of the glycolysis pathway in FG fish. Accordingly, the mitochondrial oxidative phosphorylation showed a lower activity in FG turbot as suggested by down-regulation of ADP-ATP translocase 2 (p-val = 0.051, FC = −0.64), which codes for a membrane protein responsible for transporting ATP from the mitochondria to the cytosol.

**Figure 2:**
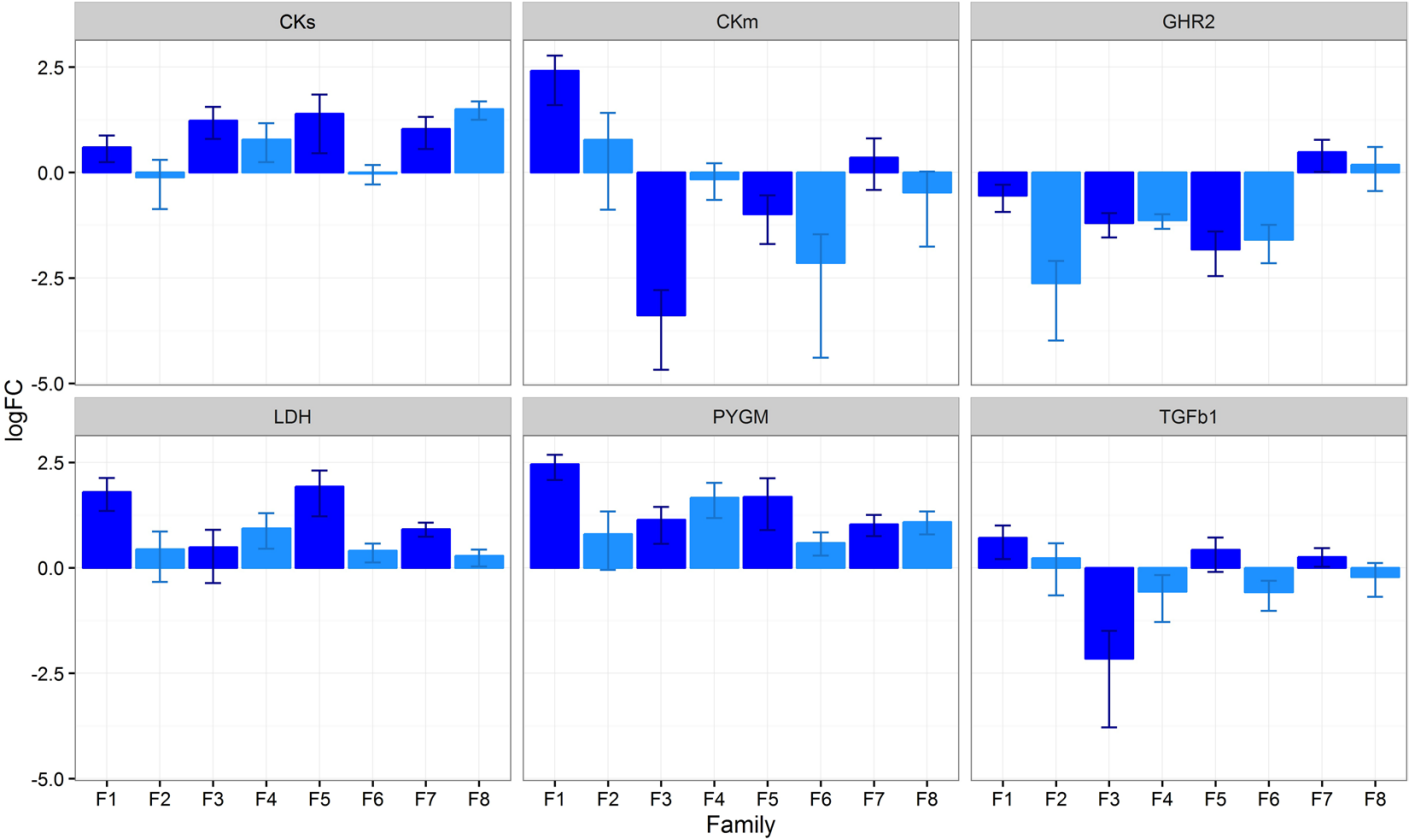
Family expression differences for six candidate genes. Log2 fold change differences between fast- and slow-growing fish in each family for six candidate genes studied by qPCR. Positive fold changes indicate over expression in fast-growing fish. The genes are lactate dehydrogenase (LDH), muscle glycogen phosphorylase (PYGM), growth hormone receptor 2 (GHR2), cytosolic creatine kinase (CKs), mitochondrial creatine kinase (CKm), and transforming growth factor β1 (TGFb1).

The most interesting genes detected in the family pools were evaluated in individual samples by qPCR: CKs, CKm, GHR2, lactate dehydrogenase (LDH), muscle glycogen phosphorylase (PYGM) and transforming growth factor β1 (TGFβ1). Although a notable concordance was observed between individual qPCR and pooled RNA-Seq data when comparing the expression pattern of FG and SG fish, some discrepancies were detected (Fig. 2), suggesting that some mechanisms driving growth might be different among families. Specifically CKm and TGFβ1 showed important variation among families. In fact, when individual qPCR data were pooled following the same distribution of individuals as for RNA-Seq, correlation was positive and significant between both datasets, except for CKm and TGFβ1 (Supplementary Table 1).

A lower number of DE genes were detected in the brain and not so straightforwardly related to growth as in the case of muscle. Among these, we observed down-regulation of phosphorilase kinase alpha 2 (p-val = 0.172, FC = −0.49) encoding for a subunit of the phosphorylase b enzyme, which is expressed in liver and brain. This gene modulates glycogen catabolism and plays an important role in supplying cell energy^22^. Also, we detected several up-regulated genes associated with sensory control, such as t-box brain protein 1 (p-val = 0.088, FC = 0.61), a transcription factor required for normal brain development and expressed in the olfactory bulb among other brain regions^23^; synaptoporin (p-val = 0.039, FC = 0.66), a vesicular transport protein involved in synaptic vesicles and plasticity^24^; and olfactomedin (p-val = 0.190, FC = 0.46), a secreted glycoprotein involved in regulating chemosensory cilia in olfactory neurons and optic nerve extension^25^. KEGG pathway and GO enrichment did not reveal any significant term.

### Differential SNP allelic frequencies (DAfreq)

**Figure 3:**
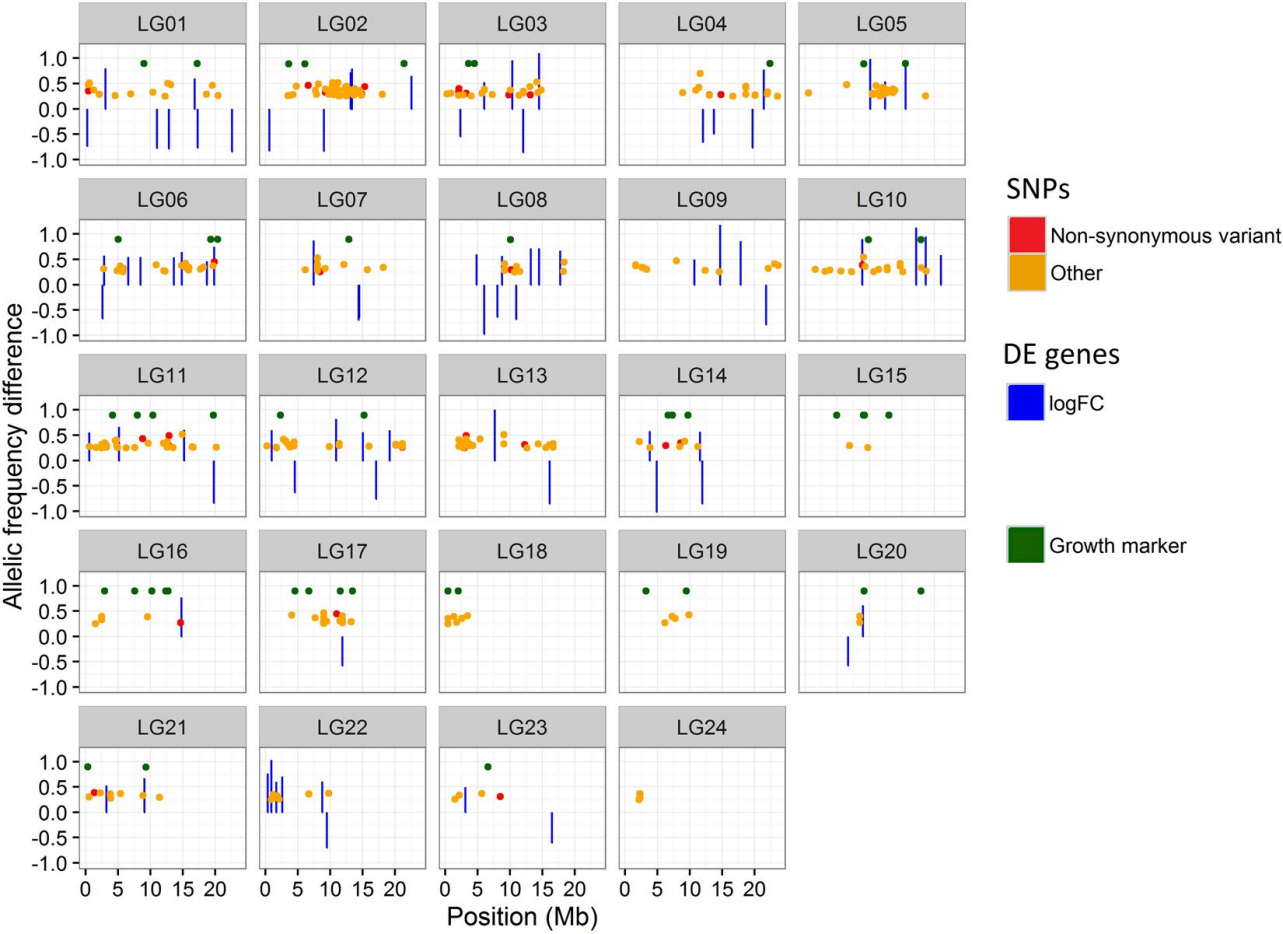
SNPs with differential frequencies along the turbot genome. SNPs showing allelic frequency differences between fast- and slow-growing turbot are shown in red (non-synonymous mutations) or orange (any other type of mutation) in the different chromosomes of the turbot genome. The fold change of those genes with FDR corrected p-values <0.3 are shown in blue and the position of genetic markers previously found associated with growth traits are shown in green.

A total of 146,217 SNPs were called using both muscle and brain reads. The sequences containing these SNPs were annotated according to the turbot genome information^21^. A total of 45,348 SNPs (31%) were found in coding regions, and among these, 16,133 corresponded to non-synonymous mutations, representing an overall Ka/Ks ratio of 0.55. Allelic frequencies were estimated for FG and SG fish and the significance of allelic divergence tested (DAfreq onwards). Significant DAfreq between FG and SG were detected for 75 SNPs in muscle and for 358 in brain (Supplementary Dataset 2). These 435 DAfreq SNPs identified were located in 326 genes: i) 43% in untranslated regions (3′ and 5′ UTR); ii) 49.2% in coding regions of annotated genes, including mostly synonymous (74%), but also missense variants (24%), and 2% located in splicing regions; iii) 3.4% in introns, and iv) 4.4% in non-annotated genes. The genomic position of these SNPs in the turbot genome^21^ and their co-localization with previously described growth-associated markers^17,18^ was evaluated (Fig. 3). DAfreq SNPs were widespread throughout the genome, although some specific regions at particular linkage groups (LG) of the turbot genetic map showed a higher concentration (i.e. LG02, LG05 or LG13), and some of them co-localized with previously reported growth associated markers. We also checked for the correspondence between DAfreq SNPs and DE genes in the turbot map (Fig. 3). A selection of the most suggestive SNPs is shown in Table 3. Several DAfreq SNPs co-localized in a narrow region of LG2, with two glycolysis up-regulated genes in muscle, glyceraldehyde-3-phosphate dehydrogenase and triosephosphate isomerase, including a synonymous SNP variant of the gene glycogen synthase kinase 3, which is involved in the negative regulation of skeletal muscle growth (Table 3; Supplementary Dataset 2). Highly significant SNPs showing a large DAfreq (>0.4) were also observed in two expressed genes in brain coding for sensory-related vesicle proteins, synaptotagmin 1 and synaptophysin, mapping at LG11 and LG16, respectively. Further, the muscle up-regulated deoxynucleoside triphosphate SAMHD1 gene showed a missense mutation located at LG10.

**Table 3:** Selection of SNPs showing divergent allelic frequencies between high and low growth turbot.

Chr	Pos	V1/V2	DAfreq RNA-seq (−log p-val)	DAfreq SNaPshot	Gene	Annotation
LG01	12,679,184	G/A	0.51 (6.8)	0.11	insulin receptor	Synon
LG02	13,982,806	T/C	0.32 (8.2)	0.15^**/s^	glycogen synthase kinase 3	Synon
LG02	15,305,418	A/T	0.44 (6.9)	0.13	glutamate receptor 2	Missense: Tyr453Phe
LG04	11,572,319	T/A	0.70 (7.8)	0.08	Cklf-like marvel transmembrane domain-containing protein 3	3′UTR
LG06	19,857,348	T/A	0.45 (9.5)	—	myosin heavy chain, fast skeletal muscle	Missense: Thr40Ser
LG10	8,837,360	T/G	0.39 (9,7)	0.30^*/**^	deoxynucleoside triphosphate SAMHD1	Missense: Lys151Gln
LG11	14,899,119	A/G	0.52 (7.2)	0.21^*/s^	synaptotagmin 1	Intron
LG16	9,519,616	C/A	0.39 (7.9)	0.05	synaptophysin protein 1	5′UTR
LG24	2,244,489	A/G	0.37 (7.8)	0.07	glucose-6-phosphatase 3	Synon

For each SNP the chromosome (Chr), the position in the chromosome (Pos), the major variant (V1), the minor variant (V2), the allelic frequency difference (DAfreq) and Fisher’s test −log10 p-value (−log pval) obtained from the RNA-seq, the DAfreq and significance of genotypic/allelic divergence (G-tests; s: p < 0.10, *p < 0.05, **p < 0.01) obtained from SNaPshot, the gene the SNP belongs to (Gene) and the SNP annotation regarding the gene (Annotation; Synon: synonimous) are shown.

Except for the SNP corresponding to fast myosin heavy chain protein, all the SNPs assayed were technically feasible and could be characterized using the SNaPshot methodology (8 out of 9). These SNPs were successfully genotyped in all FG and SG samples, and significant frequency differences for three novel candidate genes were individually validated (Table 3; Supplementary Table 2). Worth noting is the DAfreq observed for SAMHD1, which also presented differential expression between extreme growth phenotypes in this study.

## Discussion

Transcriptomes of fast- (FG) and slow- (SG) growing turbot involving eight different families from a breeding population of Atlantic origin were compared in this study. Several genes showing differential gene expression (DE) and/or divergent allelic frequencies at SNP markers (DAfreq) were found when comparing both phenotypic groups. The use of pools of families represents a cost-effective strategy for a preliminary exploration of the most important factors involved in differential growth. This approach enables identifying candidate genes, pathways and genomic regions associated with differential growth rates, as recently reported in other aquaculture species^26–29^. This strategy was here applied for the first time in the turbot, the most important farmed flatfish worldwide^30^. We identified relevant candidate genes and genomic regions associated with differential growth in this species, essential for understanding the mechanisms underlying phenotypic variation of this complex trait and useful for genetic assistance in breeding programs. The transcriptome profiles at 270 dpf here obtained also contribute key information to be further compared with other life-cycle stages with different weight of hyperplasia and hypertrophy into myogenesis^5^. Some candidate genes were individually validated using qPCR in FG and SG fish in turbot families. Aggregated individual qPCR results were mostly in agreement with RNA-Seq pools; however, some genes showed inter-family transcriptomic differences, suggesting that functional strategies associated with growth rate in turbot may be diverse among families, as previously reported for other complex traits in teleosts^31^. Our results are consistent with previous growth-association studies in turbot and other fish, where some genomic regions showed association across families, while others were family specific^17,18,32^. Furthermore, SNaPshot assays validated the association of some growth-related SNP variants identified through differential allele frequencies between FG and SG pools in the transcriptomic analysis. These gene markers could be useful for studies in other families and/or populations, or even for genome editing approaches for those potentially functional variants.

Up-regulation of all glycolytic enzymes along with lactate dehydrogenase was consistently observed in our study, which supports a higher oxidation rate of glucose by fermentation in muscle to increase growth rate. Unlike other vertebrates, fish muscle is separated in discrete layers of different fibre types. Vertebrates have two main types of striated muscle fibres, red and white, which in fish specialize in low speed swimming and bursts of maximum speed, respectively^33^. Red muscle fibres (less than 10% of the myotomal musculature) mainly depend mainly on aerobic, mitochondria driven, energy metabolism^34^. On the contrary, the metabolism of white muscle fibres (never less than 70% of fish skeletal muscle) relies mainly on anaerobic glycolysis, aided by the intracellular energy shuttle of cytosolic creatine kinase (CKs)^35^. The higher expression of glycolytic enzymes, lactate dehydrogenase (LDH) and CKs in fast-growing turbot strongly suggests a higher white muscle metabolic activity, which would presumably explain their higher growth. Glycolysis has also been found up-regulated in the muscle of domestic rainbow trout compared to their wild counterparts^36^, and also in faster-growing fish of the same species; this up-regulation has been associated with an increased muscle energy demand in fast-growing animals^29^. However, we cannot discriminate cause and consequence from our results. Either high growth rate demands an increasing anaerobic energy metabolism or, conversely, higher activity of glycolytic enzymes, LDH and CKs, results in higher growth rate. Further work is needed to clarify this issue.

Two suggestive candidates for follow-up experiments are the glycolytic enzymes triosephosphate isomerase 1 (TPI) and glyceraldehyde-3-phosphate dehydrogenase (G3PDH). Both genes were up-regulated in turbot muscle and they are closely linked in LG2 (181 kb; ~0.3 cM^21^). Further, these two genes lie within the confidence interval of a growth-related QTL in turbot^17,19^, in the vicinity (<500 kb; ~1 cM^18^) of several DAfreq SNPs. TPI markers have been found associated with length variation in other fish^37^, and its expression has also been correlated with body size, muscle fat content and growth potential in bulls^38^, as also observed for G3PDH in fast-growing rainbow trout^29^. Other clusters of a few DE genes in short genomic stretches (<1 Mb) were observed in our study, suggesting positional clustering of co-expressed genes of related pathways as reported for Arctic char^31^. The integration of structural and functional data provides clues for understanding the genetic architecture underlying growth in turbot and fish in general. The co-localization of several DE genes with DAfreq SNPs and growth-related QTL suggests that multiple genes could be underneath these growth-related genomic regions in turbot, as observed for complex traits in other fish^31^.

A higher activity of the glycolytic pathway was also supported by the up- and down-regulation of cytosolic (CKs) and mitochondrial (CKm) creatine kinase, respectively, in the muscle of turbot. Creatine is used as an energy reservoir in its phosphorylated form, and CK is responsible for creatine phosphorylation, capturing available cellular energy; but it can also catalyse the reverse reaction, using phosphocreatine to regenerate ATP, acting as a metabolic regulator^39^. While CKs activates glycolysis by removing the ATP generated, CKm is coupled to the mitochondrial oxidative phosphorylation capturing the ATP generated^40,41^. In this context, the up-regulation of CKs observed in FG turbot is likely consistent with the higher anaerobic metabolism in the white muscle. Both CKs and CKm genes have been reported to be highly regulated in muscle between large and small rainbow trout, associated with reactive oxygen stress imbalance^29^.

Growth hormone receptor 2 (GHR2), a fish-specific paralogous gene, was found down-regulated in the muscle of six out of eight turbot families, a similar expression pattern to that observed in fast-growing rainbow trout^29,41^. Growth hormone pathways are crucial for vertebrate growth, and specifically the variation at GHR2 has been also associated with growth in different tilapia species^42^. GHR2 is involved in fish myogenesis and compensatory growth^43^, although further functional analyses are required to understand its signal transduction mechanisms. Interestingly, GHR2 is located within the confidence intervals of a QTL explaining up to 18% of the phenotypic variance for body weight and length at LG14 in turbot^17^.

Unlike muscle, only a few DE genes were detected in brain when comparing FG and SG turbot. Detection of DE genes could be hindered by sampling the whole brain instead of dissecting specifically the hypothalamus or hypophysis, a very difficult task before the onset of sex growth dimorphism in turbot. Even so, to our knowledge, this is the first transcriptomic study showing brain differences between FG and SG in turbot. Interestingly, some up-regulated genes had been previously related to sensory control, including t-box brain protein 1, synaptoporin and olfactomedin, the latter two associated with domestication behaviour and feeding preference in other fish, and related to higher growth rate^44,45^. DAfreq SNPs were also found in two relevant genes coding for vesicle proteins, synaptophysin, associated with behavioural variation in domesticated zebrafish^44^, and synaptotagmin 1, related to memory formation and retention, and feeding response in fish^46^. Moreover, these SNPs mapped within the intervals of growth QTL explaining more than 10% of observed variance^17^, where major candidate genes are also located (lumican at LG16 and myogenin at LG11, respectively)^19^. Synaptotagmin 1 SNP was validated using SNaPshot, showing significant and suggestive differences between FG and SG fish at genotypic and allelic level, respectively. All these data suggest that sensory-related genes and pathways regulating learning and memory in brain might explain differences in feeding behaviour and, thus, be involved in turbot growth rate.

Numerous DAfreq SNPs were detected in muscle and brain transcriptomes distributed across all chromosomes of the turbot genome, even in linkage groups such as LG9 and LG22, where growth QTLs have not been previously reported^21^. Although many of them may reflect linkage disequilibrium with causal polymorphisms or be false positives, others may explain growth differences in turbot, especially if they co-localize with previously reported QTL, or better, if they are within candidate genes showing differential expression. Thus, this list represents a catalog of candidate markers affecting gene expression and/or protein function with small to moderate effects associated with growth. Some differential SNPs concentrated in genomic regions co-localizing with previous growth markers reported in turbot^21^, particularly focused on those found across different families, which may represent the QTL variation in the wild Atlantic population of origin^17,18^. For example, a cluster of differential variants were detected in LG12, close to 3/9CA15 explaining up to 9% of length variance; or in LG16, close to Sma-USC-E11, explaining up to 25% of Fulton’s factor variance^17^, and apparently conserved in different fish species^21^. The largest accumulation of DAfreq SNPs is observed in LG2 where, as previously mentioned, there are also two glycolitic enzymes showing differential expression. One of the SNPs in these regions is located in glycogen synthase kinase 3 (GSK3). This SNP has been validated individually and shows significant genotypic frequency differences between FG and SG fish. GSK3 is involved in the negative regulation of muscle growth and differentiation^47^. A decrease in the activity of this protein is positively related to muscle growth. Further molecular and biochemical analysis are needed to determine if the identified SNP has an impact on GSK3 and consequently on the differential growth rates.

DAfreq SNPs within DE genes represent the most robust evidence for causality. In this study, DAfreq SNPs were often found in the vicinity of DE genes, but rarely mapped within DE genes. Two noteworthy exceptions were missense variants in the gene SAM and HD domain containing deoxynucleoside triphosphate (SAMHD1) located in linkage group LG10 and the myosin heavy fast skeletal gene located at turbot LG6. SAMHD1 was found to be up-regulated and its non-synonymous SNP individually validated, being significant at allelic (p = 0.034) and genotypic (p = 0.008) level. This gene encodes a major regulator of dNTP reservoir in the cell, playing an essential role in cell-cycle progression and cell proliferation, with impact on viral replication and cellular DNA polymerization^48,49^. SAMHD1 dNTP reduction has been associated with a reduction of the number of mitochondria per cell by impeding mtDNA replication^49^. This is consistent with down-regulation of CKm in fast-growing turbot. Furthermore, this gene mapped within the confidence interval of a suggestive QTL for growth in turbot^18^. On the other side, the myosin heavy fast skeletal gene was in the vicinity (<500 kb; ~1 cM) of a growth QTL marker^18^ and was up-regulated together with other myofibrillar component genes, as also reported in fast-growing rainbow trout^29^. These observations support their interest as growth candidates for future studies.

## Conclusion

The transcriptome comparison of fast- and slow-growing turbot from eight full-sib unrelated families enabled the identification of genes showing differential expression and SNP association with growth across the turbot genome. Sound candidate genes and genomic regions emerged as potential biomarkers of differential growth in turbot. Up-regulation of the glycolytic pathway in fast-growing turbot was consistent with a lower mitochondrial activity presumably related to higher proportion of white muscle fibres. A handful of sensory-related genes showing differential brain expression or divergent allelic frequencies suggest differential behaviour related to feeding between growth phenotypes. Nevertheless, family transcriptome variability of particular genes suggests that heterogeneous mechanisms might also be involved in differential growth rate, in accordance with the polygenic nature of this complex trait. Future studies should aim to expand these findings in other developmental stages to better comprehend growth differences in turbot. Nonetheless, all genomic resources gathered here have been useful for validating differential genetic variation between growth phenotypes and will aid in further comparative studies of growth in fish, providing a set of markers with an effect in turbot growth to be evaluated in future studies.

## Methods

### Families and sampling

**Figure 4:**
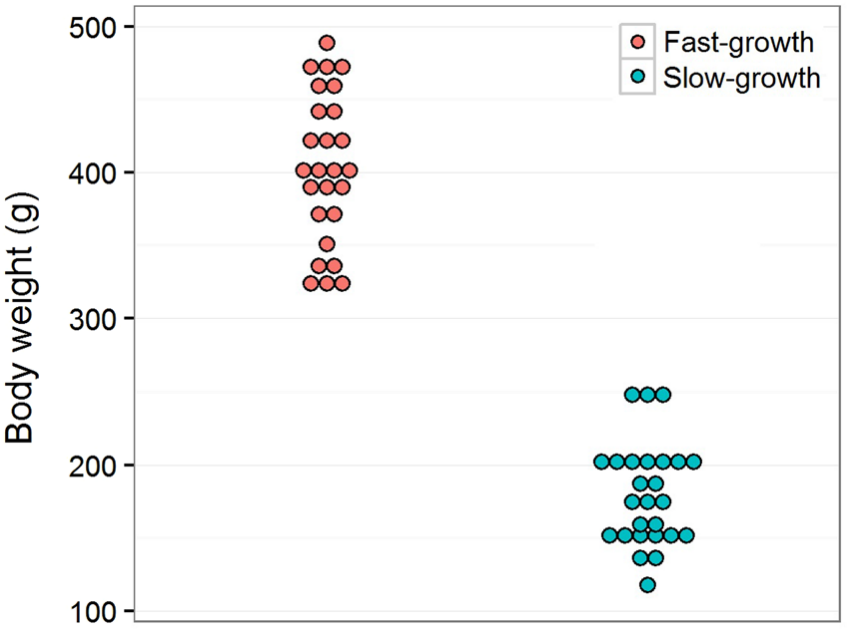
Sampling design. Body-weight distribution of fast- (FG) and slow- (SG) growth sampled groups.

Turbot from eight unrelated full-sib families coming from a pedigreed breeding strain of Atlantic origin were reared in tanks at 18 °C in the facilities of CETGA (Aquaculture cluster of Galicia; Ribeira, Spain). Fish were weighed at 270 days post fertilization (dpf), before significant differences in growth rate between males and females occured^50^. The 3–4 largest and smallest fishes from each family were sampled, rendering a total of 26 individuals for each phenotype: Fast- (FG; average weight = 402 g, SD = 51.38) and slow (SG; average weight = 180 g, SD = 35.65) growing turbot (Fig. 4; Supplementary Dataset 3). We genotyped all animals using a sex-linked molecular tool^51,52^ to identify putative sex-associated growth differences using contingency chi-square tests (p < 0.05). Muscle and brain tissues were collected and individually conserved in RNAlater (Qiagen, Valencia, CA). Animals were treated according to the Directive 2010/63/UE of the European Parliament and of the Council of 22 September 2010 on the protection of animals used for experimentation and other scientific purposes. Experimental protocols were approved by the Institutional Animal Care and Use Committee of the University of Santiago de Compostela (Spain).

### RNA sequencing

RNA extraction was performed using the RNeasy mini kit (Qiagen) with DNase treatment following manufacturer’s instructions. RNA quality and quantity were evaluated in a Bioanalyzer (Agilent Technologies, USA) and in a NanoDrop® ND-1000 spectrophotometer (Nanodrop Technologies, USA), respectively. Three libraries were constructed for each growth group (FG and SG) and tissue (muscle and brain), totaling 12 libraries. Eight to nine individuals from 2–3 different families were pooled in each library: 3 FG and 3 SG libraries for each tissue (Supplementary Dataset 3), every family being present in both FG and SG groups. Pooled samples were barcoded and prepared for sequencing at the Wellcome Trust Centre for Human Genetics, Oxford, where 100 bp paired-end (PE) reads were obtained on an Illumina HiSeq. 2000. The quality of the sequencing output was assessed using FastQC v.0.11.2 (http://www.bioinformatics.babraham.ac.uk/projects/fastqc/). Quality filtering and removal of residual adaptor sequences was conducted on read pairs using Trimmomatic v.0.32^53^. Specifically, Illumina adaptors were clipped from the reads, leading and trailing bases with a Phred score <15 were removed and the read trimmed if a sliding window average Phred score over four bases was less than 20. Only reads where both PE reads encompassed a length greater than 32 bp post-filtering were retained. Filtered reads were mapped to the recently assembled turbot genome^18^ using Tophat2 v.2.0.12^54^ that leverages the short read aligner Bowtie2 v.2.2.3^55^ with default parameters. Cufflinks v.2.2.1^56^ was used to build gene transfer format (GTF) files and cufflinks gffread utility was employed to obtain final fasta files for both muscle and brain transcriptomes. Both transcriptomes were annotated against the NCBI non-redundant protein database (nr) using BLAST v.2.2.28+^57^.

### RNA-Seq differential expression

Uniquely mapped PE-reads were counted and assigned to genes using FeatureCounts^58^ included in the SourceForge Subread package v.1.5.0. Count data were used to estimate differential gene expression between FG and SG samples using the Bioconductor package DESeq. 2 v.3.2^59^ in R v.3.2.2^60^. Genes with Benjamini-Hochberg false discovery rate (FDR) corrected p-values < 0.05 were considered differentially expressed (DE) genes. Due to the low amount of significant DE genes, those showing FDR corrected p-values < 0.3 (standard p-values < 0.005 and 0.0005 for muscle and brain, respectively) were also explored to identify a wider list of candidate genes for investigating enriched functions and pathways. Log2 library-size normalized counts were used for generating heatmaps using “aheatmap” function in R/NMF^61^. Kyoto Encyclopedia of Genes and Genomes (KEGG) pathway enrichment was assessed using KOBAS 2.0^62^ and Gene Ontology (GO) terms using Blast2GO^63^, the DE genes were compared to the whole muscle or brain transcriptomes and those terms or pathways showing FDR corrected P-values < 0.05 were considered enriched.

### Differential allelic frequencies

Pileup files describing the base-pair information at each genomic position were generated from the alignment files using the mpileup function of Samtools^64^ discarding those aligned reads with a mapping quality < 20 and those bases with a Phred score < 20. SNP calling required a minimum read depth of 50 and a minimum of 5 reads for the least frequent allele. Differential allele frequency (DAfreq) between FG and SG samples were obtained using PoPoolation2^65^ and the significance of DAfreq between the two groups was tested by Fisher’s exact test. Those SNPs showing Bonferroni corrected p-values < 0.05 and further DAfreq > 0.25 were considered significant. All the SNPs were functionally annotated using SnpEff v.4.2^66^ and the turbot genome protein annotation^21^. SNP physical location was compared with that of previously reported growth-associated QTLs in turbot^17,18^, based on other farmed families sharing an Atlantic origin with the CETGA families here studied. All these families come from broodstocks derived from the turbot Atlantic area that shows very low genetic differentiation^16^.

### Real-Time PCR

The same RNA samples used for RNA-Seq were analysed individually by qPCR. Primers for selected genes (LDH, PYGM, GHR2, CKs, CKm, and TGFβ1) were designed using the Primer 3 software^67^ (Supplementary Table 3). To determine DE between conditions the ΔΔCT method was used with the slow-growth (SG) condition set as normalizer, and the ribosomal protein S4 (RPS4) and ubiquitin (UBQ) as housekeeping genes. These two genes were previously validated for gene expression determination by qPCR in turbot^68^. Reactions were performed using a qPCR Master Mix Plus for SYBR Green I No ROX (Eurogenetec) following the manufacturer instructions, and qPCR was carried out on a MX3005 P (Agilent Technologies). Analyses were performed using the MxPro software. A t-test was used to determine significant differences between conditions. Two technical replicates of each sample were included.

### SNP validation

PCR and SNaPshot primers for SNPs included in selected candidate genes were designed using the NCBI primer designing tool and blast checked against the nr database^69^. Primer length was modified to allow multiplexing for nine SNPs showing DAfreq (Supplementary Table 4). Total DNA was purified after protein precipitation (5 M NaCl) with freezing cold absolute ethanol (1 mL). Motility rate for each potential single-base extension product was calculated using SnaPshot Primer Focus kit (Thermo Fisher Scientific) and samples were divided into two multiplex reactions and one singleplex (Supplementary Table 4). A sample of DNA from each individual was amplified and the SNaPshot minisequencing reaction was carried out using ABI Prism SNaPshot Multiplex kit (Applied Biosystems) in an ABI Prism 3730 DNA sequencer according to manufacturer’s instructions. The genotyping analysis was performed using GeneMapper ID software (Applied Biosystems). Exact G-tests were used to determine the significance of genotypic and genic differentiation between FG and SG population groups using Genepop 4.2^70^.

## Electronic supplementary material

Supplementary Info File

Supplementary Dataset 1

Supplementary Dataset 2

Supplementary Dataset 3
