# Supplementary material for: Differential gene expression and SNP association between fast- and slow-growing turbot (Scophthalmus maximus)
Source: Sci Rep. 2017 Sep 21;7:12105. doi: 10.1038/s41598-017-12459-4 (PMC5608734; doi:10.1038/s41598-017-12459-4)
Supplement: Supplementary file 1 — Supplementary Info File [file 41598_2017_12459_MOESM1_ESM.pdf]

Differential gene expression and SNP association between fast- and slow-growing turbot (*Scophthalmus maximus*)

Diego Robledo[1,2], Juan A. Rubiolo[1], Santiago Cabaleiro[3], Paulino Martínez[1], Carmen Bouza[1,*]

[1] Departamento de Zooloxía, Xenética e Antropoloxía Física, Facultade de Veterinaria, Universidade de Santiago de Compostela, 27002 Lugo, Spain

[2] The Roslin Institute and Royal (Dick) School of Veterinary Studies, University of Edinburgh, Midlothian EH25 9RG, UK

[3] Cluster de Acuicultura de Galicia (Punta do Couso), Aguiño-Ribeira 15695, Spain

[*]Corresponding author: mcarmen.bouza@usc.es; Tel./Fax: +34-982822428

Excel documents for Supplementary Datasets 1-3 are available as separate files.

Supplementary Figure 1. Glycolytic pathway enzyme-coding genes found up-regulated in turbot muscle and their fold change.

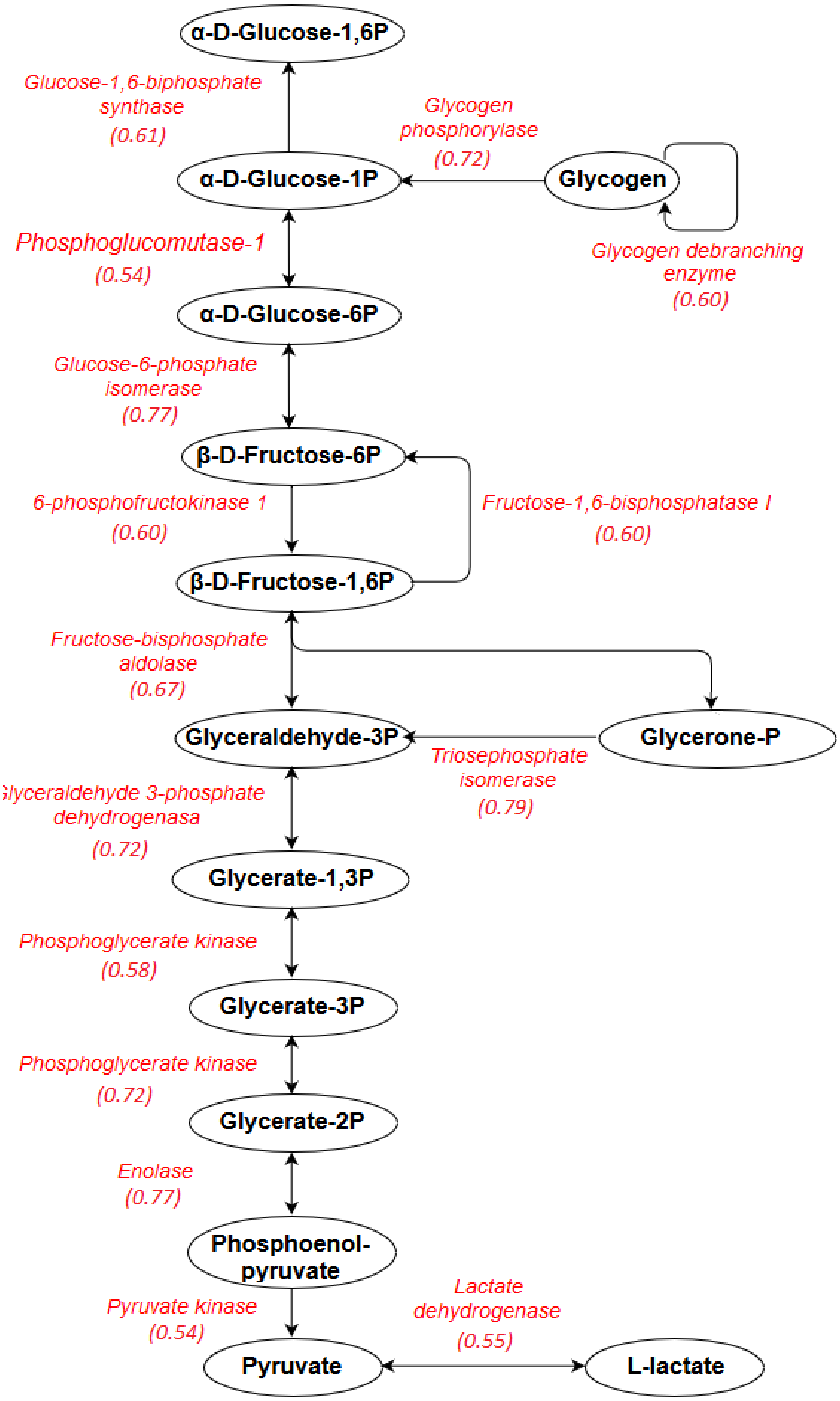

Supplementary Table 1. RNA-Seq and RT-qPCR results obtained for selected growth-related transcripts in turbot

	RNA-Seq		qPCR								
	FG *vs* SG		FG *VS* SG (Pooled Families)		FG (unpooded) *vs* SG (unpooled)						
						FG *VS* SG_group1		FG *VS* SG_group2		FG *VS* SG_group3	
**Gene Name**	**Log2 FC[1]**	**P value**	**Log2 FC**	**Conf. Int. 95%[2]**	**FG_GROUP**	**Log2 FC[1]**	**Conf. Int. 95%[2]**	**Log2 FC[1]**	**Conf. Int. 95%[2]**	**Log2 FC[1]**	**Conf. Int. 95%[2]**
Creatine Kinase-m	-0.595<br>-0.466	0.000338934<br>0.003091596	-0.6634	-0.3674 | -1.8428	FG_G1	1.3750 *	1.6973 | 0.7886	-0.6022	-0.2319 | -1.8811	-0.8424	-0.3876 | -1.8386
					FG_G2	1.6825 *	1.9713 | 1.2358	-0.2931	0.0533 | -1.4226	-0.5299	-0.0885 | -1.4087
					FG_G3	0.4100	0.7448 | -0.1931	-1.5660 *	-1.1860 | -2.8639	-1.8037 *	-1.3425 | -2.8142
Creatine Kinase-c	0.668428	0.001505129	0.8094 *	0.84124 | 0.5376	FG_G1	0.9292 *	1.0609 | 0.7369	0.3689 *	0.4526 | 0.1559	0.0707	0.3220 | -0.2323
					FG_G2	1.6573 *	1.7951 | 1.3869	1.0994 *	1.1932 | 0.8151	0.7939 *	1.0479 | 0.4203
					FG_G3	1.1517 *	1.3240 | 0.8836	0.5925 *	0.7347 | 0.2083	0.2912	0.5613 | -0.0740
TGFβ1	-0.724<br>-0.557	0.003362231<br>0.004350885	-0.4204	-0.1937 | -0.9522	FG_G1	-0.8931	-0.3354 | -1.7401	0.0064	0.2059 | -0.1880	-0.5441	-0.3162 | -1.0402
					FG_G2	-0.6923	-0.1374 | -1.5712	0.2050	0.3770 | -0.0345	-0.3458	-0.1400 | -0.8733
					FG_G3	-0.9321	-0.3702 | -1.7882	-0.0292	0.2033 | -0.2357	-0.5793	-0.3234 | -1.0833
PYGM	0.717	0.000180384	1.2452 *	1.3281 | 0.8638	FG_G1	1.8625 *	1.9722 | 1.5277	1.0330 *	1.1533 | 0.6198	0.6509 *	0.8633 | 0.2199
					FG_G2	1.8594 *	2.0277 | 1.4741	1.0271 *	1.2061 | 0.5671	0.6439 *	0.8893 | 0.1670
					FG_G3	2.1288 *	2.2991 | 1.7809	1.2938 *	1.4733 | 0.8743	0.9094 *	1.1548 | 0.4750
GHR2	-1.013	0.000198749	-1.3102 *	-1.1052 | -2.0203	FG_G1	-0.5656 *	-0.3948 | -1.0469	-1.3615 *	-1.1284 | -1.8436	-2.2343 *	-1.7442 | -3.1082
					FG_G2	-0.2747	-0.0268 | -0.6891	-1.0835 *	-0.7955 | -1.7566	-1.9723 *	-1.4680 | -3.1229
					FG_G3	-0.5207 *	-0.3645 | -0.7495	-1.3110 *	-1.0879 | -1.7793	-2.1771 *	-1.6892 | -3.0405
LDH	0.541	0.001156074	0.8411 *	0.9472 | 0.4035	FG_G1	1.2533 *	1.4590 | 0.9072	1.0260 *	1.2116 | 0.8190	-0.0298	0.2204 | -0.3025
					FG_G2	1.3525 *	1.5493 | 1.0001	1.1250 *	1.3007 | 0.9101	0.0696	0.3130 | -0.2103
					FG_G3	1.4326 *	1.7280 | 0.5977	1.2054 *	1.4891 | 0.4992	0.1489	0.4728 | -0.2634

* Significant statistical differences determined for ddCts $p < 0.05$

1 FC: fold change

2 Confidence interval

Supplementary Table 2: SNaPshot validation of the allele frequency differences between fast- and slow-growing turbot. A) p-values and significance of the genotypic and allelic differentiation ; B) Graphical representation of allele frequency differences between SNaPshot and RNAseq pool results

A)

Gene	Genotypic G-test p-value	Allelic G-test p-value
SAMHD1	0.0341	0.0079
SYT1 synaptotagmin 1	0.0386	0.0532
GSK3 glycogen synthase kinase 3	0.0094	0.1063
IR insulin receptor	0.4046	0.4095
SYP1 synaptophysin protein 1	0.8241	0.8346
GRM2 glutamate receptor 2	0.1599	0.1702
CMTM· Cklf-like marvel transmembrane domain-containing protein 3	0.4833	0.4921
G6PC3 glucose-6-phosphatase 3	0.6849	0.6705

B)

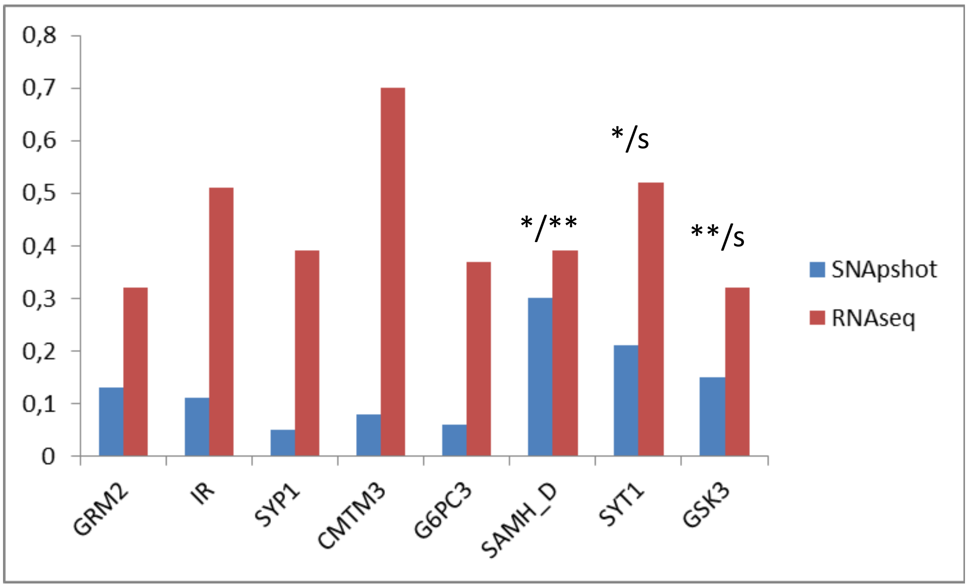

P-value: genotypic/allelic significance: (s) suggestive <0.10, (*) 0.05, (**) 0.01

Supplementary Table 3. Primers used for real-time PCR

Gene	Primer Forward	Primer Reverse
LDH	ACATCGTCAAGTACAGCCCC	GCTTGACGGGTGGATGTTGA
PYGM	GTCGTCTTGCTGCCTGTTTC	CCTCTTCAACCTGCCAACCA
GHR2	TTCAACGAGAACCACACGGA	AGTTGAGGTTGACCGGAGGA
CKs	TCTGGGCTACATCCTGACCT	ACGCCATCAACCACCATCTG
CKm	AATGAGGAGGACCACACCAG	AATGAGGAGGACCACACCAG
TGFβ1	TCGCTTCCCGTTTCATCACT	CCATGCTTTGCTCATTCCCG

Supplementary Table 4: Primers used for the SNapShot analysis

	**Forward**[1]	**Reverse**[1]	**SNaPshot**[12]
		**Multiplex 1**	
**Insulin receptor IR**	ACAGTGGACTCTTTGGCGTG	CTTGTTCCAAACCACCGTCC	gactgaACGGTGACCACTGTCTTCAG
**Glutamate receptor GRM2**	GGGAACGACCGTTTCGAAGG	TGTGAAGCAGACAATGTGAAGT	gactgactgaTCACATCCTGGGCTTCACGT
**Myosin heavy chain MHC**	TTCTTAGCCTTGTCCTCTGC	CATTGAAATCAAGCATTTGGTGACA	TGGATCTGGACCAGGTCA[3]
**Synaptophysin protein 1 SYT1**	AAATGTGAAGCCACGCCCAT	CGTCCACGACGAGGTAAAGG	gactgactgacTAAGTAGATTCAGACATGTATGG
		**Multiplex 2**	
**Cklf-like-marvel-transmembrane domain-containing protein 3 CMTM**	GGAGGAACTGGATCCTCTGTA	CAGAATCGTCTGAGACACTGAAT	gactgactgaAGCATTAAAGGGTGGAAACACA
**SAMHD1**	GATGTCTCTGCGGGTGATGA	TTCACTGTTTCAGGGTGTGCT	gactgactgactgacTACTTGGCAGGACAGCTTGTA
**Synaptotagmin 1 SYT1**	CCAAATACAAGCTGGGCTCC	GGGATGGTCGTATTTTTGCCA	gactgactgactGTAATTATGTGTCTTTATGTGTATAGTT
**Glucose-6-phosphatase 3 G6PC3**	CGTCTGTTTCCTCAGGCAGT	AGAACAGGTAGGGCGAGACT	GGTGTCCTCGCTGGGTTC
		**Singleplex**	
**Glycogen synthase kinase 3 GSK3**	CAGGTTCTGGGGACACCAAC	TGTTTAACGGGAACCCAGCA	gactTGTGGGAATTTGAACTCTGTGTA

1 all primers are in 5'→ 3'.
2 in lower case are added sequences that allowed multiplexing.
3 in red, failed SNaPshot primer for minisequencing.
